# Lack of effects of typical and atypical antipsychotics in DARPP-32 and NCS-1 levels in PC12 cells overexpressing NCS-1

**DOI:** 10.1186/1477-5751-9-4

**Published:** 2010-06-19

**Authors:** Bruno R Souza, Karen CL Torres, Débora M Miranda, Bernardo S Motta, Estêvão Scotti-Muzzi, Melissa M Guimarães, Daniel S Carneiro, Daniela VF Rosa, Renan P Souza, Helton J Reis, Andreas Jeromin, Marco A Romano-Silva

**Affiliations:** 1 Laboratório de Neurociências, Departamento de Saúde Mental, Faculdade de Medicina, Universidade Federal de Minas Gerais, Av Alfredo Balena 190, 30130-100, Belo Horizonte, MG, Brazil; 2 Departamento de Farmacologia, instituto de Ciências Biológicas, Universidade Federal de Minas Gerais, Av. Antônio Carlos, 6627, 31270-901, Belo Horizonte, MG, Brazil; 3 Banyan Biomarkers, Inc., Alachua, FL 32615, USA

## Abstract

**Background:**

Schizophrenia is the major psychiatry disorder, which the exact cause remains unknown. However, it is well known that dopamine-mediated neurotransmission imbalance is associated with this pathology and the main target of antipsychotics is the dopamine receptor D_2_. Recently, it was described alteration in levels of two dopamine signaling related proteins in schizophrenic prefrontal cortex (PFC): Neuronal Calcium Sensor-1 (NCS-1) and DARPP-32. NCS-1, which is upregulated in PFC of schizophrenics, inhibits D_2 _internalization. DARPP-32, which is decreased in PFC of schizophrenics, is a key downstream effector in transducing dopamine signaling. We previously demonstrated that antipsychotics do not change levels of both proteins in rat's brain. However, since NCS-1 and DARPP-32 levels are not altered in wild type rats, we treated wild type PC12 cells (PC12 WT) and PC12 cells stably overexpressing NCS-1 (PC12 Clone) with antipsychotics to investigate if NCS-1 upregulation modulates DARPP-32 expression in response to antipsychotics treatment.

**Results:**

We chronically treated both PC12 WT and PC12 Clone cells with typical (Haloperidol) or atypical (Clozapine and Risperidone) antipsychotics for 14 days. Using western blot technique we observed that there is no change in NCS-1 and DARPP-32 protein levels in both PC12 WT and PC12 Clone cells after typical and atypical antipsychotic treatments.

**Conclusions:**

Because we observed no alteration in NCS-1 and DARPP-32 levels in both PC12 WT and Clone cells treated with typical or atypical antipsychotics, we suggest that the alteration in levels of both proteins in schizophrenic's PFC is related to psychopathology but not with antipsychotic treatment.

## Background

Schizophrenia is the major psychiatry disorder with prevalence of approximately 1% of worldwide population [1]. It is characterized by psychosis, apathy and social withdrawal, and cognitive impairment, which results in altered functioning in many aspects of life. It is a life-long disorder and, although exact disease cause remains unknown, it is known that the disease can be triggered by a combination of genetic and environmental factors [2].

It is well known that dopamine-mediated neurotransmission imbalance is associated with schizophrenia [3-5] and several studies have demonstrated altered activity of prefrontal cortex (PFC) of schizophrenics during hallucinations, delusions and cognitive tests [6-8]. Recent studies have demonstrated change in the expression of two proteins involved with dopaminergic signaling modulation in the schizophrenics PFC [9-11]. It was reported decrease of *dopamine and cyclic adenosine 3':5'-monophosphate-regulated phosphoprotein of relative molecular mass 32,000 *(DARPP-32) and upregulation of Neuronal Calcium Sensor-1 (NCS-1) expression [12,13].

Dopamine receptors are G protein-coupled receptors classified into two subtypes: D_1_-like receptor subtypes (D_1_, D_5_), positively coupled to adenylyl cyclase (G_s_), and D_2_-like receptor subtypes (D_2_, D_3_, D_4_), negatively coupled to adenylyl cyclase (G_i_) [14]. D_1 _receptor subtype, when activated, enhances phosphorylation of DARPP-32 at threonine 34 (Thr34) by protein kinase A (PKA) [15,16] which is counteracted by the action of D_2 _receptors [17]. DARPP-32, phosphorylated at Thr34, inhibits protein phosphatase-1 (PP-1), acting as a key downstream effector in transducing dopamine signaling, integrating the signaling of different neurotransmitters and neuromodulators [18].

Desensitization and internalization of a receptor is a process that reduces cell responsiveness to neurotransmitters [19]. Dopamine D_2 _receptor internalization is regulated by G-protein-coupled receptor kinase 2 (GRK2). GRK2 phosphorylates D_2 _receptor, triggering the receptor sequestration by arrestin [20]. However, NCS-1, which is a member of EF-hand superfamily, forms a complex with GRK2 and D_2 _receptors, inhibiting this receptor phosphorylation and consequently, inhibiting its internalization [21]. Recently, it was demonstrated the colocalization of NCS-1 and D_2 _receptors in pre and post-synaptic structures of pyramidal neurons and interneurons in primate prefrontal cortex (PFC) [22].

Antipsychotics are drugs used in pharmacological treatment to diminish symptoms of schizophrenia. Because of their differences in receptor affinities and side effects, they are classified as typical and atypical. Typical antipsychotics, such as haloperidol (HAL), are D_2 _antagonists with strong affinity and slow dissociation kinetics from receptor, which is frequently associated with extrapyramidal effects [2,23]. Atypical antipsychotics, such as clozapine (CLO) and risperidone (RIS) show reduced affinity to D_2 _and are antagonists of serotonin receptors. Due to these properties, lower levels of extrapyramidal effects are observed in treatments with atypical antipsychotics [23]. Although it is well known that antipsychotics modulate schizophrenia symptoms, the molecular and biochemical mechanisms implicated in this improvement is not well established.

Because of the functions of both DARPP-32 and NCS-1 in dopaminergic signaling, the main target of antipsychotics, and their alterations in PFC of schizophrenia patients, we studied the effects of typical and atypical antipsychotics in expression of DARPP-32 and NCS-1 in five regions of rat's brain: prefrontal cortex, hippocampus, striatum, cortex and cerebellum. We did not observe any alterations in both DARPP-32 and NCS-1 expression levels after chronic treatment with antipsychotics [24]. However, one of the limitations of our study was that fact of all the animals were wild type. Since PC12 cells are commonly used as a dopaminergic model, in order to investigate involvement of NCS-1 in dopaminergic intracellular signaling pathways, we established PC12 cell line overexpressing NCS-1 by stable transfection (PC12 Clone). We observed downregulation of DARPP-32 in PC12 Clone cells (Souza, submitted). Thus, our purpose was to study if upregulation of NCS-1 modulates the effects of typical and atypical antipsychotics in the expression of DARPP-32 and NCS-1. For this, we chronically treated PC12 cells wild type (PC12 WT) and PC12 cells Clone cells with antipsychotics and investigated the levels of proteins by western blot.

## Results

### NCS-1 levels in PC12 WT and PC12 Clone cells treated 14 days with antipsychotics

**Figure 1:**
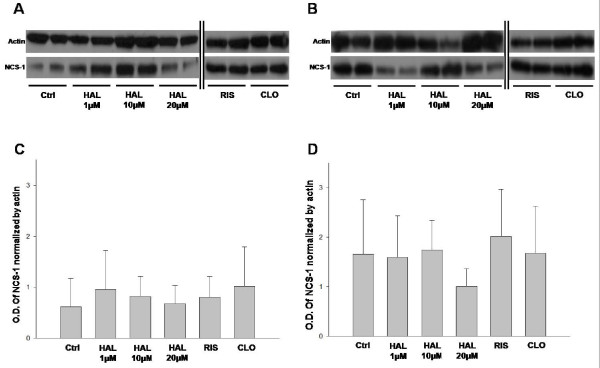
**Chronic typical and atypical antipsychotics treatments effects in NCS-1 levels**. Western blot (*A-B*) and densitometric (*C-D*) analyses of NCS-1 in PC12 WT and PC12 Clone cells treated HAL (1, 10 and 20 μM), CLO (10 μM) and RIS (20 μM) for 14 days [n = 5-7 per group]. Densitometric data of NCS-1 were normalized by Actin. The results show that there are no alterations in NCS-1 protein expression levels followed by 14 days antipsychotic administration in PC12 WT [*One Way ANOVA; *P = 0.919] and PC12 Clone cells [*One Way ANOVA; *P = 0.936]. Data are means ± SD, *p < 0.05, One Way ANOVA. HAL, Haloperidol (1, 10 and 20 μM); CLO; Clozapine (10 μM); RIS, Risperidone (20 μM).

First we confirmed that NCS-1 levels were higher in untreated PC12 Clone cells [Mean = 1.658] than untreated PC12 wt cells [Mean = 0.614] [*Student t-test*; N = 7; P = 0.044]. To address if typical or atypical antipsychotics change NCS-1 levels in PC12 WT and PC12 Clone cells, we treated them with HAL (1, 10 and 20 μM), CLO (10 μM) and RIS (20 μM) for 14 days. PC12 cells were prepared to examine NCS-1 protein expression after drug treatment and it was observed no changes in NCS-1 levels in both PC12 WT (Figure 1A and 1C) [*One Way ANOVA; *P = 0.919] and PC12 Clone cells (Figure 1B and 1D) [*One Way ANOVA; *P = 0.936] after chronic treatment with either typical or atypical antipsychotics. These results suggest that normal and upregulated levels of NCS-1 are not modulated by chronic antipsychotic treatments.

### DARPP-32 levels in PC12 WT and PC12 Clone cells treated 14 days with antipsychotics

**Figure 2:**
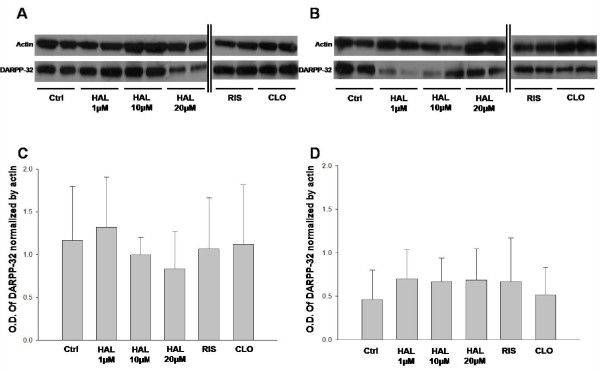
**Chronic typical and atypical antipsychotics treatments effects in DARPP-32 levels**. Western blot (*A-B*) and densitometric (*C-D*) analyses of DARPP-32 in PC12 WT and PC12 Clone cells treated HAL (1, 10 and 20 μM), CLO (10 μM) and RIS (20 μM) for 14 days [n = 5-7 per group]. Densitometric data of NCS-1 were normalized by Actin. The results show that there are no alterations in DARPP-32 protein expression levels followed by 14 days antipsychotic administration in PC12 WT [*One Way ANOVA; *P = 0.901] and PC12 Clone cells [*One Way ANOVA; *P = 0.919]. Data are means ± SD, *p < 0.05, One Way ANOVA. HAL, Haloperidol (1, 10 and 20 μM); CLO; Clozapine (10 μM); RIS, Risperidone (20 μM).

First we verified the DARPP-32 levels in PC12 Clone cells. We observed that DARPP-32 levels were decreased in untreated PC12 Clone cells [Mean = 0.459] comparing with PC12 wt cells [Mean = 1.164] [*Student t-test*; N = 7; P = 0.024]. To address if typical or atypical antipsychotics change DARPP-32 levels in PC12 WT and PC12 Clone cells, we treated them with HAL (1, 10 and 20 μM), CLO (10 μM) and RIS (20 μM) for 14 days. PC12 cells were prepared to examine DARPP-32 protein expression after drug treatment and it was observed no changes in DARPP-32 levels in both PC12 WT (Figure 2A and 2C) [*One Way ANOVA; *P = 0.901] and PC12 Clone cells (Figure 2B and 2D) [*One Way ANOVA; *P = 0.919] after chronic treatment with either typical or atypical antipsychotics. These results also suggest that normal and downregulated levels of DARPP-32 are not modulated by chronic antipsychotic treatments.

## Discussion

Drugs that target dopamine D_2 _receptors are commonly used in the treatment of schizophrenics [2], which is an evidence of the involvement of dopaminergic signaling pathway in this disorder. However, because of several studies suggest that there are no changes in D_2 _receptor expression in brains of schizophrenic patients [25], it was postulated that changes in receptor-associated signaling complex and second messengers might be involved in the dopamine disturbance in these patients [9,25].

NCS-1 inhibits phosphorylation of dopamine D_2 _receptor by GRK2 and consequently D_2_-Arrestin-GRK2 complex formation, which is responsible for internalization and desensitization of D_2 _[26-28]. Also, it was reported a colocalization of NCS-1 and D_2 _in PFC of primates [29]. Since it was previously shown that NCS-1 is upregulated in PFC of schizophrenic subjects [12,13], and that there was no alteration in NCS-1 levels in brain of rats chronically treated with typical or atypical antipsychotics [24], we hypothesized if upregulation of NCS-1 could be modulated by antipsychotic treatment. Thus, we investigated the levels of NCS-1 in PC12 Clone cells, which overexpressed NCS-1, after 14 days of typical or atypical antipsychotics treatments. We observed that there was no alteration in the levels of NCS-1 in PC12 wt and PC12 Clone cells after chronic antipsychotic treatment (Figure 1).

Recently, it was showed that levels of DARPP-32, a key downstream effector in transducing dopamine signaling, is decreased in PFC of schizophrenia subjects [10,11] and that there are DARPP-32 genetic variations associated with PFC cognitive functions [30]. However, we did not find alterations in the expression of DARPP-32 in brain of rats after chronic treatment with typical or atypical antipsychotics [24]. In a previous study we demonstrated that DARPP-32 levels are donwregulated in PC12 cells overexpressing NCS-1 (Souza, submitted). Thus, because the rats used were wild type, we addressed if the downregulation of DARPP-32 could be modulated by antipsychotic treatments. Therefore, we treated PC12 Clone cells with typical or atypical antipsychotics for 14 days. We observed that there was no change in the levels of DARPP-32 in both PC12 wt and PC12 Clone cells after chronic antipsychotic treatment (Figure 2).

## Conclusion

**Figure 3:**
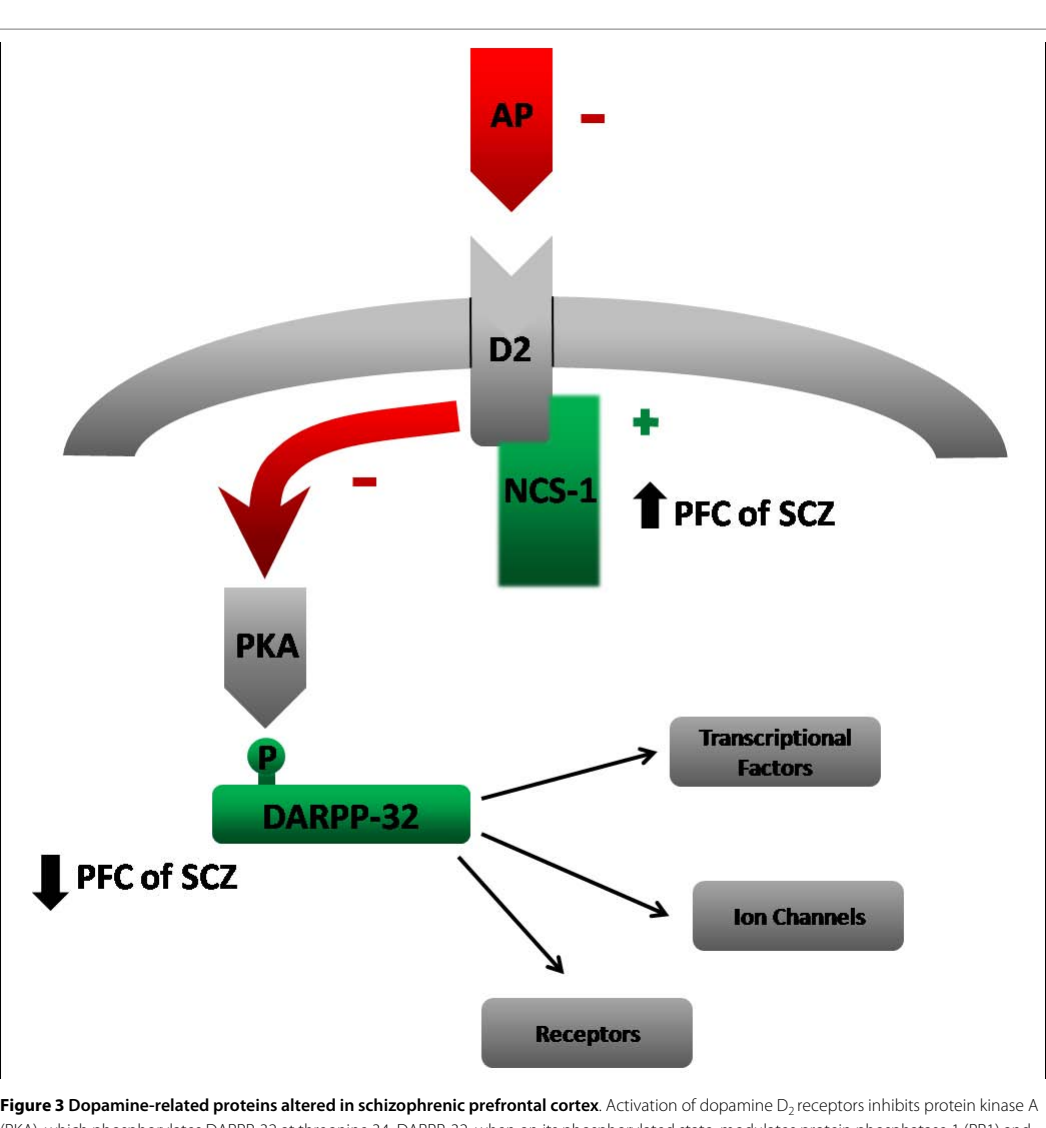
**Dopamine-related proteins altered in schizophrenic prefrontal cortex**. Activation of dopamine D_2 _receptors inhibits protein kinase A (PKA), which phosphorylates DARPP-32 at threonine 34. DARPP-32, when on its phosphorylated state, modulates protein phosphatase 1 (PP1) and consequently transcriptional factors, ion channels and receptors. NCS-1 inhibits D_2 _internalization increasing its activation by dopamine. NCS-1 is upregulated and DARPP-32 is reduced in the prefrontal cortex (PFC) of schizophrenics (SCZ). D_2 _receptor is the main target of antipsychotics. We demonstrated that levels of both NCS-1 and DARPP-32 are not modulated by typical and atypical antipsychotics. Our results suggest that these alterations are involved in the psychopathology but not in the treatment.

We previously demonstrated that levels of NCS-1 and DARPP-32 are not altered in brains of rats chronically treated with typical and atypical antipsychotics [24]. However, one of the limitations of the study was the fact of all rats were wild type. Thus, we used PC12 cells overexpressing NCS-1, which we observed a decreased levels of DARPP-32 (Souza, submitted), to investigate if there was alteration in levels of both NCS-1 and DARPP-32 protein expression after 14 days treatment with typical or atypical antipsychotics. We demonstrated that the levels of both proteins are not modulated by antipsychotics in PC12 Clone cells. Therefore, our findings reinforce the suggestion that both downregulation of DARPP-32 and upregulation of NCS-1 reported to occur in the PFC of schizophrenia patients, might be associated with the psychopathology of the disorder but not with antipsychotic treatment (Figure 3). Taking into consideration that there is no good model able to mimic the major characteristics of schizophrenia, this is an idea difficult to be tested. Thus, only an extensive investigation of intracellular integrators and modulators will shed more light on the signaling mechanisms involved in this serious psychiatric disorder and its treatment.

## Methods

### Cell culture and treatments

PC12 cells were maintained *in vitro *using high glucose DMEM supplemented with penicillin/streptomycin (100 U/mL), 5% fetal bovine serum and 5% horse serum. Cells were cultured at 37°C in a humidified 95% air/5% CO_2 _incubator. The medium and drugs were replaced every 2 days and the passages were performed every seven days. PC12 cells overexpressing NCS-1 were grown in DMEM as described above with addition of G418 (400 mg/mL - Clonetch). Reagents used for cell culture were purchased from Invitrogen Corporation (USA). PC12 cells stable overexpressing NCS-1 were obtained as described by Koizumi [31]. PC12 WT and PC12 Clone cells were treated for 14 days with 1, 10 and 20 μM Haloperidol (Sigma - H1512), 10 μM Clozapine (Sigma - C6305) and 20 μM Risperidone (Sigma - R3030).

### Immunoblot

PC12 (wt and Clone) cells lysates were sonicated and centrifuged at 13,000 × g for 30 min at 4°C. Supernatants were transferred to plastic tubes, protein was quantified and extracts stored at -80°C. 50 μg of each sample was prepared for electrophoresis with sample buffer NuPAGE LDS (Invitrogen) plus 10% of β-mercaptoethanol and incubated at 70°C for 10 min. The samples were loaded into bis-Tris NuPAGE 4-12% gels (Invitrogen) and submitted to electrophoresis followed by transfering to nitrocellulose membranes (Hybond ECL, Amersham Pharmacia Biotech). Protein loading and efficiency of blot transfer were monitored by staining with Ponceau S (Sigma Chemical Co., USA). The membranes were blocked for 45 min with PBS Tween 20 0.1% plus 5% non-fat milk. Membrane blots were incubated with polyclonal anti-NCS-1 antibody (1:2000 - FL-190, Santa Cruz Biotecnology), polyclonal anti-DARPP-32 (1:250 - H-62, Santa Cruz Biotechnology) and monoclonal anti-actin antibody (1:5000 - MAB1521R - Chemicon) diluted in PBS Tween 20 0.1%, for 2 hours at RT. Then, membranes were washed and incubated for one hour at RT with horseradish peroxidase (HRP)-conjugated secondary antibodies, goat anti-rabbit IgG (1:20000) and goat anti-mouse IgG (1:7000) (secondary antibodies were purchased from Molecular Probes). Membranes were submitted to chemiluminescent detection with ECL Plus (Amersham Biosciences) as described by manufacturer, and visualized on ImageQuant. Densitometric analysis was performed using Scion Image Software version Beta 4.0.2 (Scion Corporation, National Institutes of Health, USA).

### Statistical Analysis

All data are presented as means ± Standard Deviation of the Mean (SD). Differences among experimental groups in experiments evaluating protein expression were determined by *Student t-test *and *one-way ANOVA*. In all experiments, P values lower than 0.05 were considered to significant.

## Competing interests

The authors declare that they have no competing interests.

## Authors' contributions

BRS, BSM, ESM, MMG and DSC performed the experiment. BRS, DVFR and RPS analyzed the data. BRS, KCL, DMM and HJR wrote the manuscript. AJ developed a new research tool. MARS conceived the study. All authors read and approved the final manuscript.
